# Mycotoxin exposure is associated with increased risk of esophageal squamous cell carcinoma in Huaian area, China

**DOI:** 10.1186/s12885-019-6439-x

**Published:** 2019-12-16

**Authors:** Kathy S. Xue, Lili Tang, Guiju Sun, Shaokang Wang, Xu Hu, Jia-Sheng Wang

**Affiliations:** 1 Department of Environmental Health Science, College of Public Health, University of Georgia, 150 Green Street, Athens, GA 30602 USA; 2 Southeast University School of Public Health, Nanjing, Jiangsu China; 3 Huaian District Center for Disease Control and Prevention, Huaian, Jiangsu China

**Keywords:** Aflatoxin B_1_, Co-exposure, Fumonisin B_1_, Esophageal cancer, Molecular epidemiology, Mycotoxins

## Abstract

**Background:**

Consumption of moldy food has previously been identified as a risk factor for esophageal squamous cell carcinoma (ESCC) in high-risk countries; however, what contributing roles these dietary carcinogenic mycotoxins play in the etiology of ESCC are largely unknown.

**Methods:**

A mycotoxin biomarker-incorporated, population-based case-control study was performed in Huaian area, Jiangsu Province, one of the two high-risk areas in China. Exposure biomarkers of aflatoxins (AF) and fumonisins (FN) were quantitatively analyzed using HPLC-fluorescence techniques.

**Results:**

Among the cases (*n* = 190), the median levels of AF biomarker, serum AFB_1_-lysine adduct, and FN biomarker, urinary FB_1_, were 1.77 pg/mg albumin and 176.13 pg/mg creatinine, respectively. Among the controls (*n* = 380), the median levels of AFB_1_-lysine adduct and urinary FB_1_ were 1.49 pg/mg albumin and 56.92 pg/mg creatinine, respectively. These mycotoxin exposure biomarker levels were significantly higher in cases as compared to controls (*p* <  0.05 and 0.01, respectively). An increased risk to ESCC was associated with exposure to both AFB_1_ and FB_1_ (*p* <  0.001 for both).

**Conclusions:**

Mycotoxin exposure, especially to AFB_1_ and FB_1_, was associated with the risk of ESCC, and a greater-than-additive interaction between co-exposures to these two mycotoxins may contribute to the increased risk of ESCC in Huaian area, China.

## Background

Esophageal cancer is one of the most common cancers worldwide, ranked 7th in incidence in males, 6th and 9th in cancer-related death in males and females worldwide, respectively [1], and considerably higher in both incidence and cancer-related death in many developing countries [1–3]. The highest incidences of esophageal cancer were found in East and Southern Africa, Northern France, and Central Asia, with the notorious “Central Asia esophageal cancer belt” extending from northeastern China westward to northern Iran along the Silk Road, spanning several countries including China, Turkmenistan, Uzbekistan, Karakalpakstan, Kazakhstan, and Iran [2–4]. There are two main types of esophageal cancers, namely, the squamous cell carcinoma (ESCC), and adenocarcinoma (EAC). ESCC constitutes of majority of esophageal cancer cases, and was more prevalent with high incidences in developing nations such as China, with the primary risk factor being dietary patterns and environmental factors, as well as individual genetic predispositions [1, 5]. Whereas for EAC, the rates are increasing in the US and other developed nations in past decades, and the risks were postulated to be linked to obesity, as well as various preexisting conditions, such as Barrett’s esophagus and reflux symptoms [6].

Consumption of moldy and mycotoxin-contaminated food has previously been identified as a risk factor for ESCC in high-risk countries [5, 7, 8]; however, what contributing roles thesedietary carcinogenic mycotoxins play in the etiology of ESCC are largely unknown. Aflatoxin B_1_ (AFB_1_) and fumonisin B_1_ (FB_1_) are among the most well-known mycotoxins found in commonly consumed foods and feed products [9]. AFB_1_, a Group 1 human carcinogen and a representative of the aflatoxin family, is produced by fungal contaminants, *Aspergillus flavus* and *A. parasiticus* [10]. It is considered to be a major food contaminant worldwide, affecting food and products of, including but not limited to, corn, peanuts, milo, sorghum, copra, and rice [11]. FB_1_, on the other hand, is a Group 2B carcinogen and a representative of fumonisin family, produced primarily by maize pathogens, *Fusarium verticillioides* and *F. proliferatum*, which contaminates maize and maize-based products ubiquitously [12, 13]. As both *Aspergillus* and *Fusarium* can contaminate and often co-exist on maize and some other cereal grains, concerns for human co-exposure to these two mycotoxins, and its consequences, have been raised [14, 15]. Co-existence of AFB_1_ and FB_1_ in food items has already been reported in several studies worldwide, particularly from Asia, South and Central America, and Africa [16–21]. Consequently, efforts must now be made to assess the extent of human co-exposure to these mycotoxins, as well as the adverse health effects they may have, in order to more accurately assess the risk posed by the nature of co-contamination and co-exposure [22].

Dietary FB_1_ exposure has been proposed as one of the major environmental factors associated with increased risk of ESCC in developing countries [23]. The first association between FB_1_ and human esophageal cancer was proposed by Sydenham *el al*., who reported FB_1_ contamination in the Transkei region, a high incidence area of esophageal cancer in Africa [7, 23]. In Huaian, China, another high-risk area for ESCC, FB_1_ was detected in 95.7% (112/117) of corn samples, with an average of 2.84 mg/kg (range: 0.1–25.5 mg/kg) [24]. Co-contamination of FB_1_ with other mycotoxins, particularly AFB_1_, has also been reported in this high ESCC risk area. In Huaian area, the mean level of AFB_1_ in food was estimated to be 13.5 μg/kg, with estimated daily intake of 1.723 μg (range 0.224–49.772 μg) [19]. Similarly, in high risk areas of Cixian and Linxian counties, China, co-contamination of AFB_1_ and FB_1_ were detected at mean levels of 8.61 μg/kg (range: 1–38.4 μg/kg) and 35.3 mg/kg (range: 20–60 mg/kg), respectively [8].

The use of molecular biomarkers, facilitated by understanding of toxicokinetics and toxicodynamics of environmental toxicants, has improved the assessment of individual exposure beyond the levels detected in external media, such as in food, as well as estimations based on questionnaires [25]. For AFB_1_, the most widely acceptable exposure biomarker is serum AFB_1_-lysine adducts, formed by covalently binding of AFB_1_-diol with lysine residue on serum albumin [11]. It is reasonably stable in blood until albumin turnover, thus making the levels of this adduct highly reflective of chronic exposure [11]. This adduct has been validated and utilized as a reliable indicator of chronic exposure in both animal and human epidemiological studies [11, 26]. For FB_1_, the orally dosed toxin is poorly absorbed and rapidly eliminated with no major metabolites produced in humans; nonetheless, the parent compound can be measured directly in biological fluids, such as urine, as a biomarker of FB_1_ exposure, which has also been validated in human population studies [27–31].

Dietary exposure to mycotoxins has been linked to the etiology of several gastrointestinal cancer sites and chronic diseases in many developing countries [7, 9, 32, 33]. The co-contamination of AFs and FNs in human diets has been widely reported, and epidemiological studies have indicated the potential contributory effect of the two mycotoxins in etiologies of human liver and esophageal cancer [19, 20, 34, 35]. Combinative exposure to the two mycotoxins often showed greater toxic effects, and potentially synergistic effects on tumorigenesis, in animal and human cell models, when compared to either AFB_1_ or FB_1_ alone [36–38]. To better understand the contributing roles and mechanisms these exposures have in human esophageal carcinogenesis, we used biomarker approach to evaluate their contributing risk of ESCC, specifically, serum AFB_1_-lysine and urinary FB_1_. Furthermore, we examined potential interactions, including additive effects, between mycotoxin exposures and increased risk of ESCC in Huaian, China.

## Methods

### Chemicals and reagents

AFB_1_-lysine adduct standard was synthesized and purified as previously described by Sabbioni et al. [39]. Albumin determination reagent (bromocreosol purple), and normal human serum were purchased from Sigma Aldrich Chemical Co. (St. Louis, MO). Pronase (25 kU, Nuclease-free) was purchased from Calbiochem (La Jolla, CA). Protein assay dye reagent concentrate and protein standards were purchased from Bio-Rad Laboratories Inc. (Hercules, CA). Boric acid, o-phthaldialdehyde (OPA), 2-mercaptoethanol, FB_1_ from *F. verticilioides* (~ 98% purity, TLC), 10× phosphate buffered saline (PBS), ammonium hydroxide, ammonium acetate, sodium chloride, sodium phosphate monobasic, hydrochloric acid, and formic acid were purchased from Sigma-Aldrich (St. Louis, MO, USA). OPA reagents were prepared by dissolving 10 mg of OPA and 30 μl of 2-mercaptoethanol in 250 μl of methanol and mixing with 4.75 ml of 3% boric acid buffer (pH 10.5) and stored at 4 °C avoiding light before use. Mixed mode solid phase extraction (SPE) cartridges, as well as Sep-Pak reversed phase C18 cartridges were purchased from the Waters Corp. (Milford, MA). All other chemicals and solvents were of highest grade and purity available.

### Study site and populations

**Fig. 1:**
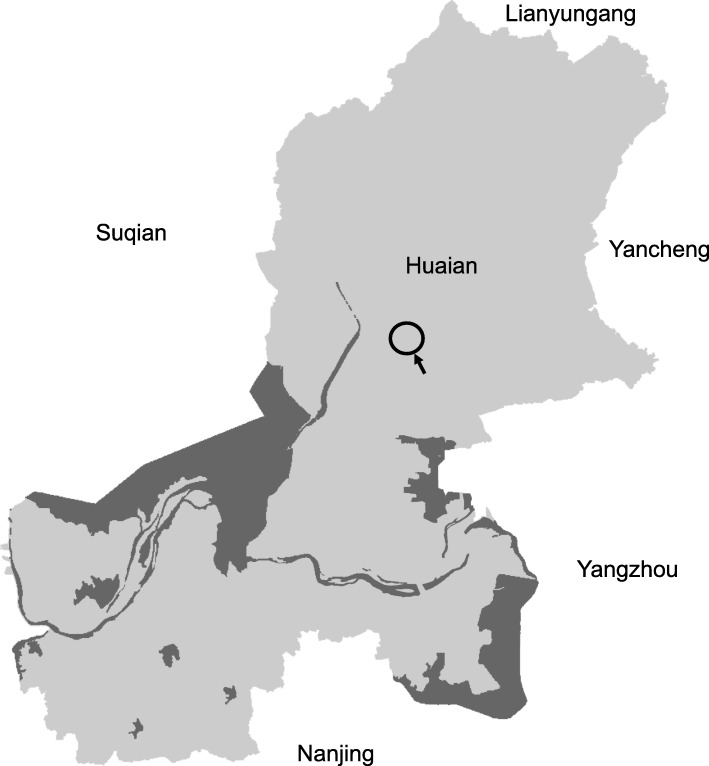
Map of Huaian area, Jiangsu Province, China. Circled with arrow indicate the townships where the study participants were recruited for this case-control study. Map of Huaian was traced using Adobe Photoshop CS2 (https://www.adobe.com/), with texts and indicators added with Microsoft PowerPoint (https://www.microsoft.com/en-us/). No copyright issue present

Huaian area, located in the northern area of Jiangsu Province of China, is one of the two endemic areas for esophageal cancers in China (the other being the southern Taihang Mountain area, including Linzhou of Henan Province and Cixian of Hebei Province), with incidence over 80 per 100,000, six times greater than the national average rate [5]. The study followed a population-based case-control design, with the participants recruited from five rural farming communities (townships) belonging to the Huaian District. The location of the study site is shown in Fig. 1. Cases consist of ESCC diagnosed in 2006–2007 from the malignant tumor registration record, and healthy controls were matched by age, gender, and residency. After signed written consent, a face-to-face interview was conducted, and a total of 190 cases and 380 controls were recruited. Questionnaire on demographics [5, 40], disease history and dietary pattern, blood sample (5 mL), and the morning urine sample (50 mL) were collected. Personnel conducting laboratory analyses were blinded to case and control status. The study protocols including ethics guideline and consent form were approved by the Institutional Review Boards for human subjects at Southeast University School of Public Health and Texas Tech University (human subject assurance number: 00001568) and was compliant with human research guidelines of the respective institutions.

### HPLC-FLD analysis of serum AFB_1_-lysine adduct

Overall sample processing used a method previously reported in Qian et al. 2013 [41]. Briefly, thawed human serum samples underwent pathogen deactivation via submerging sample tubes in 56 °C water bath for 30 min. Serum albumin and total protein were analyzed with respective reagents, as described previously. An aliquot of 150 μl serum was then digested via pronase (1:4 pronase:total protein, w:w), in 37 °C water bath for 3 h to optimize the conditions of enzyme digestion in order to release lysine adducts. The contents were then purified via solid phase extraction, using Waters MAX SPE cartridges over vacuum chamber manifold. Samples were eluted with 2% formic acid in methanol, vacuum-dried with a Labconco Centrivap concentrator, and reconstituted with 150 μl of 25% methanol prior to injection.

AFB_1_-lysine adduct was quantified using Agilent 1100 HPLC-fluorescence detection system (Agilent Technologies, Wilmington, DE, USA), at excitation/emission of 405/470 nm. Chromatographic separations were achieved using Zorbax Eclipse XDB-C18 reverse phase column (5 μm, 4.6 × 250 mm), with a gradient of 20 mM NH_4_H_2_PO_4_, pH 7.2 (Buffer A), and 100% methanol (Buffer B), to achieve separation within 25 min at flow rate of 1.0 ml/min. For each injection, 100 μl of processed samples were used. Final concentrations were obtained via manual integration and calculation using a standard curve, then adjusted by albumin content of corresponding samples, to obtain the exposure parameters in units of pg/mg albumin. The limit of detection for the method used is 0.4 pg AFB_1_-lysine /mg albumin.

### Measurement of urinary Creatinine

Creatinine concentration in urine samples was analyzed using the 96-well Creatinine Assay Kit from Cayman Chemical (Ann Arbor, MI, USA). The analysis is based on a modified Jaffe colorimetric method that measures the difference in absorbance (495 nm) of the creatinine-picrate complex before and after acidification. The assay was carried out according to manufacturer’s instructions, and absorbance was measured using an ELx808 Absorbance Microplate Reader from BioTek Instruments, Inc. (Winooski, VT, USA).

### HPLC-FLD analysis of urinary FB_1_

Urinary FB_1_ was analyzed based on previous studies [42]. Briefly, 10 ml aliquot of urine samples were centrifuged to remove pellets. The supernatant was passed through FumoniTest column by gravity. After washing with 10 mL 1x PBS, FB_1_ was eluted three times with 0.5 mL of 20% methanol in 10 mM hydrochloric acid directly onto pre-conditioned Waters Oasis HLB cartridge and sequentially washed with HPLC grade water and 25% methanol, three times each. The contents were then eluted with 2% formic acid in methanol and dried under a gentle stream of nitrogen gas while incubated in 35 °C water bath. Dried residues were then reconstituted with 200 μl 50% methanol, and 150 μl were transferred to injection vial for analysis.

HPLC analysis was performed with Agilent 1100 HPLC-fluorescence detection system. On-line derivatization of analytes with OPA reagent was performed via injection program, which mixes 10 μl of reagent with sample for 1 min prior to injection. The injection volume was 100 ul. Chromatographic separation achieved Zorbax Eclipse XDB-C18 reverse phase column (5 μm, 4.6 × 250 mm) maintained at 35 °C. The mobile phases consist of 0.1 M sodium phosphate monobasic (pH 3.4) (Buffer A) and methanol (Buffer B). The flow rate of mobile phase was 1.0 mL/min, consisting of linear gradient starting from Buffer A: Buffer B (35:65, v/v) to Buffer A: Buffer B (20:80, v/v) over 13 min. OPA derivatives of FB_1_ were monitored at excitation/emission of 300/ 440 nm. The limit of quantitation was 20 pg per injection. The measured urinary FB_1_ levels were adjusted by creatinine concentration of corresponding urine samples to obtain the exposure parameters in units of pg/mg creatinine.

### Statistical analysis

Descriptive statistics, including mean ± SD, median, and range (or distribution quadrants), were calculated for each exposure parameter. Detailed epidemiological questionnaire data, including tobacco, alcohol consumption, dietary pattern, etc., were assessed as well. The association between exposure parameters with the risk of ESCC was estimated by conditional logistic regression model. Odds ratios (ORs) and their 95% confidence interval (CI) for ESCC risk were calculated. The interaction of mycotoxins was estimated by using crossover analysis. Interaction parameters, including relative excess risk due to interaction, proportion attributable to interaction, and synergy index, were calculated using methods described by Andersson et al. [43]. All statistical tests were two-sided, with *p*-value of < 0.05 considered statistically significant. All analyses were conducted in SAS 9.4 (SAS Institute, Cary, NC, USA).

## Results

### Population characteristics

Demographic information is summarized in Table 1. There were no significant differences between cases and controls in cumulative alcohol and tobacco uses. Significant differences were found between cases and controls in education attainment, as well as individual income per month at the time of recruitment and 10 years before the recruitment.

**Table 1:** Demographic and socioeconomic characteristics of cases and matched controls

Characteristic	Cases (*n* = 190) No. (%)^c^	Controls (*n* = 380)	p^a^	OR (95% CI)^b^
Gender				
Male	111 (58.4)	222 (58.4)	1.00	1.0
Female	79 (41.6)	158 (41.6)		1.00 (0.70–1.43)
Educational attainment				
Illiterate	113 (59.5)	185 (48.8)	< 0.001	1.00
Primary school	55 (29.0)	106 (28.0)		0.74 (0.47–1.15)
Middle school	17 (9.0)	64 (16.9)		0.30 (0.15–0.60)
High school and above	5 (2.6)	24 (6.3)		0.25 (0.09–0.72)
P for trend				< 0.001
Marital Status				
Married	146 (76.8)	314 (82.9)	0.07	1.0
Not Married (divorced, widowed, single)	44 (23.2)	65 (17.2)		1.54 (0.96–2.47)
Ten-years-ago income, yuan/month				
< 50	52 (27.4)	108 (28.4)	0.01	1.0
50–100	43 (22.6)	127 (33.4)		0.73 (0.46–1.18)
≥ 100	95 (50.0)	145 (38.2)		1.44 (0.92–2.26)
P for trend				0.07
Current income, yuan/month				
< 100	56 (29.5)	75 (19.7)	0.02	1.0
100–300	82 (43.2)	175 (46.1)		0.63 (0.41–0.97)
≥ 300	52 (27.4)	130 (34.2)		0.50 (0.30–0.83)
P for trend				0.008
Mean ± SD				
Age, years	62.0 ± 7.9	61.9 ± 7.8	0.85	1.00 (0.98–1.03)
Cumulative alcohol intake (kg-year)	2.6 ± 2.4	2.9 ± 3.3	0.36	0.96 (0.87–1.05)
Cumulative tobacco use (pack-year)	39.6 ± 47.4	47.0 ± 66.5	0.25	1.00 (0.99–1.00)

^a^weighted t test of mean difference between cases and controls or conditional logistic regression of difference between cases and controls, as appropriate

^b^odds ratio (OR) and 95% confidence interval (CI) were calculated using conditional logistic regression or unconditional logistic regression with adjustment of age and gender, as appropriate

^c^percentage may not add to 100 because of rounding

### Mycotoxins exposure and ESCC risks

**Fig. 2:**
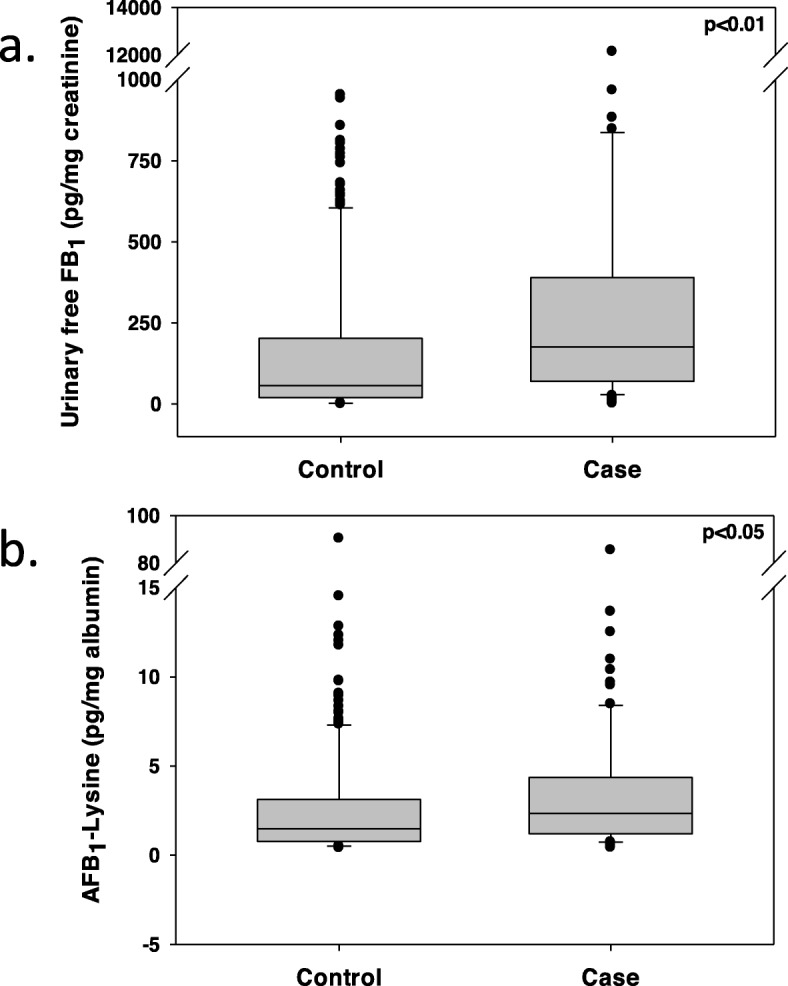
Comparison of biomarker levels between cases and controls. Plots depict overall levels of **a** urinary FB_1_ and **b** serum AFB_1_-lysine adducts in esophageal cancer case and control samples. Boxes in the plot represent 25 and 75 percentiles, with the medians as the middle lines. Bars represent 5 and 95 percentiles of data

Urinary free FB_1_ was detectable in 95.8% (181/189) of the ESCC cases and 84.2% (319/379) of the controls, and serum AFB_1_-lysine was detectable in 90.7% (146/161) of the cases and 69.3% (142/205) of the controls. The mean, median, standard deviation, and quartiles for AFB_1_-lysine as well as urinary FB_1_ for both cases and controls are summarized in Table 2 and Fig. 2. Both urinary free FB_1_ (Fig. 2a) and serum AFB_1_-lysine (Fig. 2b) levels were significantly higher in cases than in controls (*p* <  0.01 and <  0.05, respectively).

**Table 2:** Serum AFB_1_-lysine and Urinary FB_1_ levels of Huaian Esophageal Cancer Case and Control samples

		AFB_1_-lysine (pg/mg albumin)	Urinary FB_1_ (pg/mg creatinine)
Case	Median	1.77	176.13
	mean ± SD	6.03 ± 13.36	470.41 ± 1215.95
	Quartile (25 & 75%)	(1.04, 4.7)	(70.77, 388.67)
Control	Median	1.49	56.92
	mean ± SD	3.67 ± 11.12	213.56 ± 437.75
	Quartile (25 & 75%)	(0.67, 2.35)	(19.78, 202.27)
*P* value		< 0.05	< 0.01

Table 3 shows the ESCC risks associated with exposure to FB_1_ and AFB_1_. Continuous variables were converted into binary categorical variables based on the population median for urinary FB_1_ and serum AFB_1_ in control groups, thereby dividing the populations into low- and high- exposure groups. For both urinary FB_1_ and AFB_1_, high exposure groups have significantly higher odds ratios for ESCC (*p* <  0.001 for all), with greater percentage of cases categorized as high exposure for both FB_1_ and AFB_1_ (Table 4).

**Table 3:** ESCC risk and urinary free FB_1_ and serum AFB_1_-lysine levels

	Range^a^	Cases (%)	Controls (%)	OR (95%CI)	P
Urinary FB_1_ (pg/mg creatinine)					
Low	< 56.92	40 (21.05)	190 (50.0)	1	
High	≥ 56.92	150 (78.95)	190 (50.0)	3.689 (2.438–5.582)	< 0.001
AFB_1_-Lysine (pg/mg albumine)					
Low	< 1.49	64 (33.68)	194 (51.05)	1	
High	≥1.49	126 (66.32)	186 (48.95)	2.629 (1.711–4.041)	< 0.001

^a^Range of low and high levels for urinary FB_1_ and serum AFB_1_-lysine are determined by median level of control samples

**Table 4:** Interaction of FB_1_ and AFB_1_ exposure on risk of esophageal cancer

FB_1_ exposure^a^	AFB_1_ exposure^b^	Cases (%)	Controls (%)	p	OR (95% CI)
Low	Low	14 (7.36)	100 (26.32)		1
High	Low	50 (26.32)	88 (23.16)	< 0.001	4.057 (2.101, 7.834)
Low	High	25 (13.16)	90 (23.68)	0.060	1.983 (0.972, 4.048)
High	High	101 (53.16)	102 (26.84)	< 0.001	7.070 (3.791, 13.187)
Relative excess risk due to interaction (RERI)					2.030 (−0.591, 4.651)
Proportion attributable to interaction (AP)					0.287 (−0.029, 0.603)
Synergy index (S)					1.502 (0.864, 2.612)

Exposure levels are indicated by levels of urinary FB_1_^a^ and serum AFB_1_-lysine^b^

### Interaction between two mycotoxins exposures

Evaluation of interaction between FB_1_ and AFB_1_ exposure on ESCC risks is summarized in Table 4. The risk for high levels of exposure to both FB_1_ and AFB_1_ is significantly higher than that of either high FB_1_ or high AFB_1_ alone. The combination of high exposure to both FB_1_ and AFB_1_ resulted in positive interaction, indicating a potentially greater-than-additive risk with co-exposure of FB_1_ and AFB_1_ in affecting ESCC risks.

## Discussion

In the present study, we assessed the exposure to potent mycotoxins, AFB_1_ and FB_1_, using corresponding exposure biomarkers and evaluated their associations with the risk for ESCC in Huaian area, China. We evaluated risk associated with individual factors, as well as binary interaction between factors based on logistic regression. Based on the results, high level exposure to AFB_1_ and FB_1_ were associated with increased risk to ESCC, and greater-than-additive interaction exists between high AFB_1_ and FB_1_ exposure. The overall dietary patterns and staple food have not been changed for decades in the study area and populations [5, 19, 40], despite of possible periodic variations; therefore, measurement for biomarker levels are reflective of both the current and past exposures, which might be even higher than the currently measured exposure levels.

Previous studies have confirmed the association between FN exposure and ESCC risks, with the earliest association between ESCC and FN was established in the Transkei region of southern Africa, an international esophageal cancer hot spot [7, 44]. Studies have found significantly higher levels of *Fusarium verticillioides* infection and FB_1_ and B_2_ levels in homegrown corn samples collected from higher-risk areas compared to those from lower-risk areas [45–48]. Various populations within the region were also found to have exposure levels exceeding the recommended provisional maximum tolerable daily intake of 2 μg/kg bw [46, 47, 49]. In China, a survey of 282 corn samples from 6 provinces found detectable FB_1_ in 99.6% samples, with 25.2% of samples having levels higher than 5 mg/kg [50]. In high risk counties of Cixian and Linxian (now Linzhou), FB_1_ has been detected in all corn samples collected from local households, where half of the samples, with heavy mold contamination, have a mean FB_1_ level of 74 mg/kg (range: 18–155 mg/kg), while the other half, without visible mold contamination, showed an average FB_1_ level of 35.3 mg/kg (range: 20–60 mg/kg) [8]. However, a biomarkers-based study found no association of sphingosine, sphinganine, and sphinganine/sphingosine ratios with ESCC [51], though later studies suggested that sphingolipid levels may not be sensitive biomarkers for exposure in human populations [48, 49]. Although these studies suggested the potential role of FB_1_ exposure in esophageal cancer development, confirmations of FB_1_ exposure in human disease risks with validated exposure biomarkers have not been studied and published.

While AFs are more commonly associated with risk for hepatocellular carcinoma (HCC) [9], evidences have suggested their potential link to the risk of ESCC. Co-contamination of AFs with FNs has been reported in high-risk areas of ESCC in China, including Cixian and Linxian (now Linzhou), as well as Huaian regions [8, 19]. The association between AFs contamination and risks of esophageal cancer was further demonstrated by a study in Golestan province of Iran, where the total AFs levels in wheat flour samples was significantly higher in high-risk areas compared to low-risk areas [52]. The current study, which detected exposure to AFB_1_ via serum exposure biomarkers, confirms the co-contamination of AFB_1_ with FB_1_ in the high-risk area, as well as the association of high AFB_1_ exposure with ESCC risk. Furthermore, the current study found a greater-than-additive interaction between AFB_1_ and FB_1_ exposure via both binary interaction assay and higher-order analysis. It should be noted, however, that our study found marginal significance (*p* = 0.06) in terms of association of ESCC with low FB_1_-high AFB_1_ exposures. This may be due to the lack of sufficient participant numbers recruited in our study (n) to delineate the higher significance. On the other hand, the role of AFB_1_ exposure in contributing risks of cancers other than liver, such as lung, colorectal and esophagus, have been rarely examined [53, 54]. While the association between food AFB_1_ contamination and ESCC has been reported, there is a very high likelihood that co-contaminations of AFB_1_ with FB_1_ or other mycotoxins is also present in the study areas [33, 52]. While the potential mechanisms of AFB_1_ in ESCC risk remain to be elucidated, in an in vitro study, AFG_1_, a less toxic variant of AFs, is shown to reduce the expression of HLA-I, TAP-1, and LMP-2, critical components in antigen presentation and antigen processing, in adult esophageal epithelial cells, which can ultimately lead to defect in antigen presentation to T-lymphocytes, potentiating tumorigenesis via escaped immune surveillance, therefore potentially linking AF exposure to esophageal cancer [55].

Studies have demonstrated that the co-contamination of AFs and FNs, and the potential interaction of the two, may play a great role in etiology of many mycotoxin-related diseases and health outcomes. Epidemiological studies have pin-pointed the potential contributory effect of FB_1_ on HCC linked to AFB_1_ exposure, with co-contamination of AFB_1_ and FB_1_ detected in several areas with historically high risks for HCC, including parts of Guatemala and China [19, 20, 34, 35]. Using molecular biomarkers of exposure, Shirima et al. found chronical co-exposure to both AF and FN among young Tanzanian children via contaminated diet, and the levels were negatively associated with growth parameters, implying contribution to growth impairments [56, 57]. At the same time, in animal and model organism studies, combinations of the two mycotoxins often showed greater toxic effects when compared to either AFB_1_ or FB_1_ alone, with some studies pointing to a clear additive effect on toxicity, and others showing potentially synergistic effects on tumorigenesis; for example, FB_1_ was shown to promote AFB_1_-initiated liver tumor in rainbow trout and development of pre-neoplastic liver lesions in F344 rats [36–38]. The mechanisms of toxicity suggest that, while FB_1_ does not act as direct carcinogen like AFB_1_, it can be a potent promoter of AFB_1_-induced tumorigenesis, as FB_1_ can significantly alter sphingolipid profile in both animals and humans by inhibiting ceramide synthase, which can further affect apoptosis and potentially other signaling pathways [37, 38, 58–61]. Although further studies are required to fully elucidate the interaction between AFB_1_ and FB_1_ in human and animal carcinogenesis and other adverse health effects, it is clear that analysis of single mycotoxin may not be sufficient in assessment of potential cancer risks, and the setting of regulatory standards, which were established based on individual toxicant or carcinogen, may not necessarily be protective enough when considering the combinative effect of mycotoxins.

As shown in Table 1, we did not find significant association of ESCC cases with alcohol consumption and smoking status in the current study, which is consistent with many previously published studies in China, including studies for liver cancer, as well as studies conducted in the same area for esophageal cancer [5, 40, 62, 63]. The major reasons for lack of such association are the prevalence of alcohol drinking (nearly 100% in the study participants) and the nature of the matched case-control design. The same would apply for the lack of association for smoking status, with almost 100% study participants are active (males) or secondhand (females) smokers. The limitation of this study includes the nature of case-control study design, which can’t reveal a causative correlation, but only show associations.

## Conclusions

Mycotoxin exposure, especially to AFB_1_ and FB_1_, was associated with the risk of ESCC, and a greater-than-additive interaction between co-exposures to these two mycotoxins may contribute to the increased risk of ESCC in the studied population in Huaian area, China.

## Data Availability

The datasets used and/or analyzed during the current study are available from the request to the corresponding author.

## Abbreviations

AFB_1_: Aflatoxin B_1_
EAC: Adenocarcinoma
ESCC: Esophageal squamous cell carcinoma
FB_1_: Fumonisin B_1_
HCC: Hepatocellular carcinoma
